# Unna Boot Therapy Reduces Emergency Department Use in an Unhoused Patient: A Case Report

**DOI:** 10.5811/cpcem.63835

**Published:** 2026-08-31

**Authors:** Ariana Coba Clementel, Frank L. Ross

**Affiliations:** * New York University Grossman School of Medicine, New York City, New York; † New York University Grossman School of Medicine, Department of Surgery, New York City, New York

**Keywords:** wound care, people experiencing homelessness, venous leg ulcers, ED utilization, case report

## Abstract

**Introduction:**

Venous leg ulcers disproportionately affect people experiencing homelessness and contribute to recurrent emergency department (ED) use. Standard wound care protocols often require daily dressing changes and leg elevation, which are unrealistic in unstable housing environments.

**Case Report:**

A 71-year-old male with a 10-year history of venous leg ulcers and 15 ED visits achieved complete wound epithelialization after four months of weekly Unna boot compression therapy, despite eviction during treatment. He has not returned to the ED for this condition.

**Conclusion:**

Emergency department-initiated compression therapy with structured wound care referral may reduce recurrent ED use among unhoused patients with venous leg ulcers.

## INTRODUCTION

Chronic venous insufficiency is a common and morbid condition that frequently leads to venous leg ulcers, which are chronic wounds characterized by delayed healing and high recurrence rates.^1^ Venous pathology accounts for 70% of chronic lower extremity wounds, reflecting the central role of impaired venous return and sustained inflammation in the disruption of healing.^2^,^3^ Consequently, venous leg ulcers contribute substantially to patient morbidity and healthcare utilization. They are cause for substantial financial strain in developed nations, accounting for $148.65 billion in US health expenditures in 2022.^4^ Despite their prevalence, venous leg ulcers require ongoing compression therapy and reliable follow up, which are often not feasible within fragmented healthcare systems. Without reliable outpatient management, the emergency department (ED) often becomes the default site of care for patients with chronic wounds. This episodic model of care addresses acute symptoms without correcting the underlying venous pathology, contributing to recurrent visits. This pattern is especially pronounced among people experiencing homelessness, who face higher rates of peripheral vascular disease and disproportionately rely on the ED for care.^5^ Venous leg ulcers account for up to 20% of medical complaints among people experiencing homelessness, likely reflecting a combination of inadequate nutrition, limited rest, poor hygiene, prolonged standing or sitting, and restricted access to longitudinal care,^6^ and they contribute significantly to repeat ED use (1.63–18.75 times that of housed individuals).^7^ Therefore, venous leg ulcers are a frequent source of repeat ED use for people experiencing homelessness, reflecting both disease burden and a gap in accessible, sustained treatment.

Compression therapy remains the cornerstone of venous leg ulcer management, with evidence supporting its effectiveness in healing wounds through improved venous return and reduction of venous hypertension.^6^,^8^ The Unna boot, a zinc oxide–impregnated compression bandage, provides barrier protection and sustained graduated pressure. It can be maintained for up to one week, reducing the need for frequent dressing changes. This makes it a practical option for patients with limited access to consistent wound care.

Previous studies have consistently demonstrated that the Unna boot is an effective, low-cost compression therapy for venous leg ulcers, supporting its role in conservative management.^9^ Systematic reviews and randomized studies show comparable or improved healing outcomes to other compression modalities; however, data were derived from outpatient clinics and do not evaluate outcomes related to ED utilization.^10^,^11^ Despite its established use in outpatient and wound care settings, ED-initiated Unna boot therapy and impact on ED utilization remains poorly described in the literature. This represents a potential gap in care delivery for people experiencing homelessness who frequently rely heavily on episodic ED visits for management of chronic wounds.

We present a case of an unhoused individual with recurrent ED use for venous leg ulcer management, in whom consistent outpatient clinic Unna boot therapy was associated with successful wound healing and cessation of repeat ED visits. This case highlights a potential underused strategy for bridging gaps in care and reducing avoidable ED use in a high-risk population.

## CASE REPORT

A 71-year-old male with a history of hypertension, chronic obstructive pulmonary disease, asthma, obstructive sleep apnea, chronic venous insufficiency with prior iliac vein stent placement, and chronic venous leg ulcers presented to the outpatient wound care clinic with worsening ulceration of the right lower extremity. His symptoms included pruritus, erythema, mild pain, and serous drainage. The patient’s history of venous leg ulcers extended back approximately 10 years, beginning in 2015, during which time he required 15 total ED visits, for complications such as cellulitis, ulcer drainage, and severe leg pain. Over a five-year period from 2020 to 2025, he was treated intermittently by the vascular surgery service, which attributed his condition to venous insufficiency. Arterial Doppler studies ruled out arterial disease, and venous Doppler studies were negative for deep vein thrombosis. In 2020, he underwent placement of a right external iliac venous stent. Despite this, his management remained largely conservative, consisting of intermittent courses of oral antibiotic therapy, dressing changes with silver-impregnated hydrofiber dressings (Aquacel® Ag), compression bandaging, and instructions to elevate the lower extremities at home.

Because of the persistent and refractory nature of the ulcer, he was referred to the outpatient wound care clinic for further management. At his initial evaluation on October 16, 2025, physical examination revealed warm lower extremities without pallor or cyanosis, although both legs demonstrated woody edema, brown hyperpigmentation, and erythema with a maculopapular rash consistent with stasis dermatitis. Closer inspection of the right medial ankle revealed a full-thickness ulcer with a granular base and light fibrinous exudate (Image 1). The wound was slightly moist but lacked purulence, odor, or exposure of deeper structures such as bone or tendon. The ulcer measured 6 × 3 × 0.2 cm, and there was no evidence of surrounding cellulitis. Palpation of peripheral pulses was limited due to significant edema.

*CPC-EM Capsule*

What do we already know about this clinical entity?

*Venous leg ulcers are common, slow to heal, and often lead to repeat Emergency Department (ED) visits when compression therapy and follow-up are not sustained.*

What makes this presentation of disease reportable?

*An unhoused patient with years of recurrent ED use had venous leg ulcer healing and no further ED visits after consistent Unna boot therapy.*

What is the major learning point?

*Unna boot compression is a practical, low-barrier treatment for venous leg ulcers that can work even when housing instability limits ideal wound care.*

How might this improve emergency medicine practice?

*Earlier ED recognition and initiation of compression therapy may reduce repeat visits and connect high-risk patients to definitive wound care.*

Initial treatment focused on establishing a consistent wound care and compression regimen. After cleansing the ulcer with normal saline, topical silver alginate (Silvercel ™) was applied, followed by placement of a paste Unna boot extending from the base of the toes to just below the tibial tuberosity. A light layer of Coban™ was used to secure the dressing without excessive tension. The procedure was well tolerated, and the patient was instructed to return weekly for reassessment and reapplication. He was also advised to elevate his lower extremities above heart level when resting. This approach aimed to both protect the wound and provide sustained compression, thereby improving venous return and promoting ulcer healing.

Over the following weeks, the patient received consistent weekly Unna boot compression at the clinic. However, his course was complicated in early January 2026 by a significant social setback, as he reported eviction from his home and transition to living in a shelter. This change severely limited his ability to elevate his legs and maintain optimal hygiene. Despite these challenges, he continued to attend weekly wound care appointments.

By February 2, 2026—four months after initial presentation—physical examination of the right lower extremity demonstrated complete epithelialization of the ulcer, with no residual open areas, drainage, odor, or signs of infection (Image 2). Although chronic woody edema and xerosis persisted, there was no evidence of cellulitis, and the right foot remained warm and well perfused.

Ultimately, successful healing of the venous leg ulcer was achieved without complication. Notably, the patient has not required further ED visits for this condition since completing the treatment.

## DISCUSSION

This case highlights the practical utility of Unna boot compression in an unhoused patient with a venous leg ulcer, initiated in the outpatient setting and with subsequent impact on ED utilization. The patient reported difficulty ambulating, elevating his lower extremities, and maintaining basic hygiene because of access to showers and clean clothing. Standard wound care recommendations, particularly those requiring daily dressing changes and prolonged leg elevation, are therefore unrealistic for people experiencing homelessness. This population faces numerous barriers to healing, including inadequate nutrition, limited rest, restricted access to exercise and outpatient follow-up, mistrust of healthcare clinicians, and difficulty obtaining medications and pain control.^12^ Critically, people experiencing homelessness are often unable to maintain consistent leg elevation, a cornerstone of venous leg ulcer management.^13^

Unna boot compression offers a practical alternative that accounts for these constraints. The Unna boot delivers sustained, graduated compression that improves venous return while also functioning as a protective dressing, thereby reducing the need for frequent dressing changes and hygiene. By consolidating compression and wound coverage into a single application that can be maintained once weekly, this modality significantly reduces dependence on daily wound care, stable hygiene, self-management, and infectious risk.

Although therapy in this case was initiated in the outpatient setting, its subsequent implementation was associated with a termination of recurrent ED utilization. This suggests that earlier initiation of compression therapy may interrupt the cycle of repeated ED presentations. Therefore, the ED represents a critical potential point of intervention for earlier initiation of therapy. Patients with venous leg ulcers present to the ED for pain management, infectious complications, or dressing failure. Given the simplicity and speed of Unna boot application, ED initiation of therapy is feasible and could be incorporated into existing wound care workflows without significant disruption. Earlier initiation in the ED may therefore shorten the time to definitive compression therapy and reduce reliance on episodic, palliative ED care.

Within the ED, several steps may facilitate implementation. First, increased recognition of venous leg ulcers among unhoused patients is essential to ensure appropriate identification and referral. Second, emergency physicians can initiate Unna boot compression at the point of care because of its brief application time and compatibility with standard wound care workflows. Third, structured referral pathways to accessible outpatient wound care services remain critical for ongoing management. As demonstrated in a narrative review and program evaluation, community-based wound care clinics provide low-threshold, cost-effective longitudinal care, facilitate connection to social services, and reduce stigma associated with traditional healthcare settings.^14^ Together, this strategy addresses the core pathophysiology of venous leg ulcers through compression therapy while accommodating the realities of unstable housing. Early initiation in the ED may further enhance this effect by shortening time to definitive treatment and reducing episodic, fragmented care.

This report contributes to the literature by demonstrating that a low-barrier, compression-based wound care strategy initiated in the outpatient setting reduced downstream ED utilization in an unhoused patient. It also highlights the ED as a potential site for earlier initiation of therapy to prevent recurrent visits. To our knowledge, the link between timing of compression therapy and ED use in people experiencing homelessness has not been well described.

## LIMITATIONS

Although patient follow-up was limited by housing instability, the observed termination in ED use suggests meaningful downstream system-level impact. While single-case observations limit generalizability, this report supports the hypothesis that earlier initiation of compression therapy, potentially in the ED, may reduce frequent ED use for people experiencing homelessness.

## CONCLUSION

Wound care for venous leg ulcers is often poorly adapted to the realities of homelessness, contributing to recurrent ED utilization for pain control, infection, and repetitive dressing changes. In this case, Unna boot compression was associated with cessation of recurrent ED visits, despite unstable housing, limited leg elevation, inconsistent hygiene, and restricted access to nutrition and medications. Given inconsistent availability of wound care infrastructure, scalable ED-integrated strategies such as Unna boot initiation is urgently needed and tailored to people experiencing homelessness. Further study is warranted to evaluate the effectiveness of ED initiated compression therapy on wound healing and ED use.

## Figures and Tables

**Image 1:**
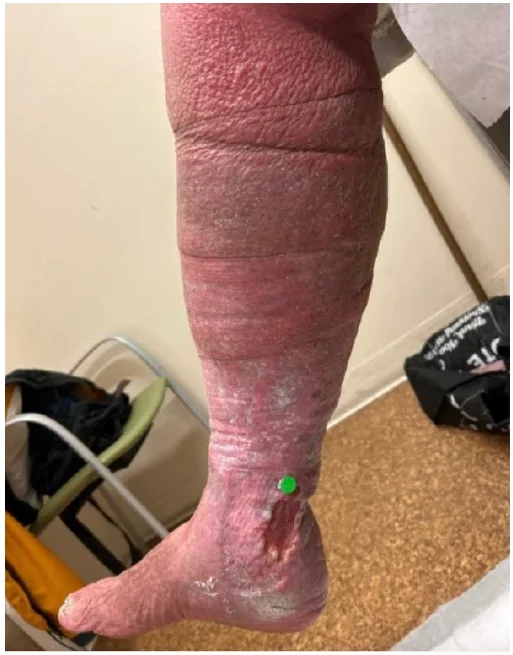
First presentation of right venous leg ulcer in a patient with chronic venous insufficiency.

**Image 2:**
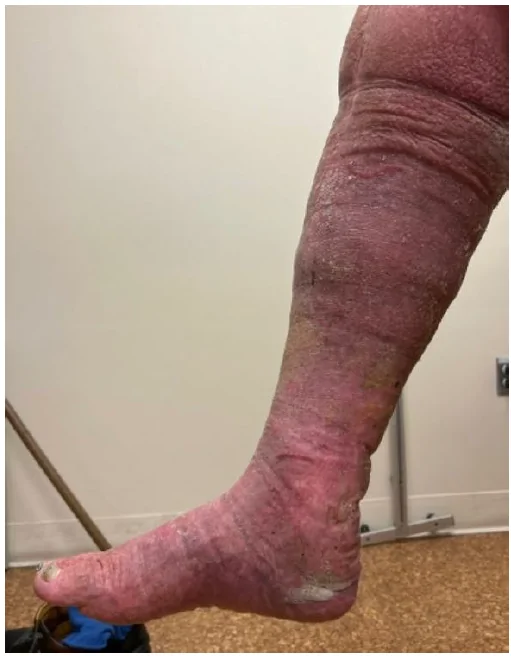
Complete epithelization of right venous leg ulcer after 15 weeks of Unna boot compression.
